# Impact of acuinjection at ST36 on leg movements, their associated respiratory events, and electroencephalographic arousals in patients with obstructive sleep apnea: A case series

**DOI:** 10.1097/MD.0000000000043401

**Published:** 2025-07-25

**Authors:** Anjyu Minami, Haruna Minami, Harumi Matsuoka, Takero Fukutome

**Affiliations:** a Certified Polysomnographic Technologist of the Japanese Society of Sleep Research, Fukuoka Sleep Clinic, Fukuoka, Japan; b Board Certified Fellow of the Japanese Society of Sleep Research, Fukuoka Sleep Clinic, Fukuoka, Japan.

**Keywords:** acuinjection, acupuncture, apnea–hypopnea index, obstructive sleep apnea, polysomnography, respiratory-related leg movement, ST36

## Abstract

**Rationale:**

Leg movements (LMs) frequently co-occur with obstructive sleep apnea (OSA), and respiratory-related LMs can trigger arrhythmias and cause diagnostic challenges during polysomnography (PSG). While the cardiovascular burden of periodic limb movements in sleep is recognized, little is known about the role of LM suppression during PSG in improving diagnostic accuracy. This case series examines the impact of acuinjection at ST36 on LM suppression during PSG in patients with OSA.

**Patient concerns:**

All 3 patients underwent diagnostic PSG due to suspected OSA. PSG was temporarily paused during acuinjection, and the PSG period was divided as follows: Pre-a (before acuinjection), Post-a (from PSG resumption to LM recurrence, defined as multiple LMs within a 5-minute period), and restarted PSG (the remaining PSG time after resumption, excluding any positive airway pressure titration if applicable). In each case, frequent LMs associated with arousals were observed during Pre-a, interfering with sleep quality and complicating the interpretation of respiratory events.

**Diagnoses:**

Initial PSG findings were suggestive of moderate to severe OSA with frequent LMs and associated arousals. Respiratory events were often temporally linked to LMs, resulting in substantial sleep fragmentation and diagnostic uncertainty.

**Interventions:**

Acuinjection was performed bilaterally at ST36 using pentazocine (3.75 mg in 0.5 mL normal saline in 1 case, and 7.5 mg in 1.0 mL in 2 cases). PSG was resumed immediately afterward.

**Outcomes:**

Outcomes were assessed using the apnea-hypopnea index (AHI), hypopnea index (HI), leg movement index (LMI), respiratory-related leg movement index (rLMI), and arousal index (ArI). In the Pre-a period (mean: 76.7 minutes), severe OSA with high LMI and ArI was observed: AHI 72.6, HI 72.3 (obstructive HI: 39.1, central HI: 33.2), LMI 141.6, rLMI 68.4, and ArI 100.0. In the Post-a period (184.8 minutes), these indices markedly improved: AHI 19.9, HI 18.9, LMI 5.9, rLMI 0.7, and ArI 36.7. During the restarted PSG (357.6 minutes), AHI, LMI, and ArI remained low at 23.4, 13.4, and 36.9, respectively. No adverse effects were observed.

**Lessons:**

This case series suggests that acuinjection at ST36 may effectively suppress LMs and associated arousals during PSG in OSA patients, thereby facilitating more accurate diagnosis and characterization of respiratory events.

## 1. Introduction

Periodic limb movements in sleep (PLMS) are recurring episodes of highly stereotyped limb movements that occur during sleep.^[1]^ PLMS complications are common in obstructive sleep apnea (OSA) and have been shown to have detrimental effects, including promoting cerebrovascular accidents.^[2]^

Periodic limb movement (PLM) is a type of leg movement (LM) that occurs 4 or more times consecutively with an inter-movement interval between ≥ 5 s or ≥ 10 seconds and ≤ 90 s.^[3,4]^ LMs associated with respiratory events, such as apnea–hypopnea, referred to as respiratory-related LM (rLM), are excluded from PLM. rLM occurs around respiratory events – the period from 0.5 to 2 seconds before a respiratory event or termination of a respiratory event to 0.5 to 10.25 seconds after the event – in patients with OSA.^[3,4]^ Respiratory events with rLM demonstrate a greater increase in heart rate than respiratory events without rLM, suggesting that increased activation of the autonomic nervous system may lead to increased cardiovascular risk.^[5]^ Thus, rLM and PLMS are strongly associated with OSA.

Guilleminault et al pointed out the role of non-ventilatory variables in the development of sleep apnea in cases of PLM.^[6]^ Therefore, in polysomnography (PSG) for OSA cases with frequent LM, the apnea–hypopnea index (AHI) may not accurately reflect the severity of OSA or the state of sleep. In this context, observing sleep and respiratory conditions under suppressed LM during PSG is reasonable when frequent LM and sleep fragmentation occur at the start of PSG for sleep apnea syndrome diagnosis.

The highest quintiles of the PLM index (PLMI; ≥65.1) are associated with significant changes in sleep architecture, including an increased percentage of stage 2 sleep and a decreased percentage of stage 3 to 4 sleep.^[7]^ Therefore, frequent LMI is approximately defined as an LMI of ≥ 60 events/h, close to 65.1, as a tentative guideline. The reevaluation of suppressed LM can, if necessary, be conducted on a separate occasion under OSA treatment.

Acuinjection at ST36 is a known method for the rapid suppression of LM during PSG.^[8,9]^ Another representative pharmacological approach for suppressing LM is oral pramipexole. However, this method requires time for the effects to manifest, making it less optimal for use during limited overnight PSG. Additionally, even if PLMs are resolved, dissociated electroencephalography (EEG) arousals from PLM^[10]^ may still influence PSG findings.

In this report, we present 3 cases of suspected OSA in which frequent LMs occurred following the start of PSG, resulting in persistent sleep fragmentation. Acuinjection at ST36 achieved nearly complete suppression of LMs. Notably, the appearance of central and obstructive hypopnea linked to rLM was also suppressed, and EEG arousals were remarkably reduced. Acuinjection at ST36 may be useful for the treatment of LMs (including rLM and PLMS) and for elucidating the underlying pathology.

## 2. Methods

According to Watanabe,^[11]^ symptoms can be alleviated by performing acuinjection with solutions such as normal saline (NS) into acupoints along the meridians passing through areas of pain or discomfort. The strength and duration of the effect depend on the solution used, with the potency ranked as narcotics > pentazocine > NS. A dose as small as 0.01 mL can yield sufficient effects.

This method was applied to 3 patients with restless legs syndrome (RLS), and acuinjection with 0.25 mL of NS into 4 acupoints on the lower limbs (GB41, BL60, ST36, and SP6) resulted in significant symptom relief (including the suppression of LMs).^[8]^ Additionally, it was suggested that single-point acuinjection at ST36 could suppress LMs.^[9]^

Subsequently, the inhibitory effects of acupoints, injection doses, and the composition of injection solutions on LMs associated with OSA were observed. The method adopted in this study was deemed reasonable based on these observations.

The study participants had suspected OSA, with frequent LMs (reference value: LMI ≥ 60) observed at the start of diagnostic PSG. Bilateral acuinjections at ST36 were performed to suppress the frequent LMs. Details of the acuinjection procedure are as follows: With PSG electrodes in place, the patient was positioned supine on the bed with the knee on the acuinjection side flexed at 90°. The practitioner identified the ST36 acupoint and administered the acuinjection. The skin over ST36 was disinfected using ethanol solution (Japanese Pharmacopoeia, 76.9 to 81.4 vol% ethanol). A sterile needle (27G, 25 mm length) was inserted perpendicular to the skin surface to a depth of approximately 1.5 cm. NS containing 3.75 mg of pentazocine in a volume of 0.5 mL was used in Case 1, while NS containing 7.5 mg of pentazocine in a volume of 1.0 mL was used in Cases 2 and 3. The acuinjection was administered slowly. The acuinjection was administered only once, with no additional injections given. The patients were instructed to report any sensory changes in the lower leg during the procedure.

During the investigation period at Fukuoka Sleep Clinic, 21 patients received acuinjections at acupuncture points (GB41, BL60, ST36, and SP6) for LM suppression. Among these, 6 patients received acuinjections exclusively at bilateral ST36 with pentazocine (3.75 mg in 0.5 mL NS or 7.5 mg in 1.0 mL NS). Of these, the following cases were excluded from the study: 2 patients who underwent positive airway pressure (PAP)-titration PSG without diagnostic PSG and 1 patient with facial nerve palsy following a cerebral infarction who was using antihypertensive drugs (including beta-blockers). The remaining 3 patients constituted the participants in this study.

Diagnostic PSG was conducted by certified PSG technologists accredited by the Japanese Society of Sleep Research. Overnight PSG, using an Alice 6 LDxS (Philips Respironics, Murrysville), took place from 10:00 pm to 6:00 am, and recorded data were analyzed using Sleepware G3 (Philips). PSG parameters (excluding LM) were primarily obtained through automated analysis and subsequently corrected manually, while rLM identification was mainly performed manually.

The PSG setup and scoring were performed according to the American Association of Sleep Medicine Manual for the Scoring of Sleep and Associated Events, Version 2.5.^[3]^ rLM-linked respiratory events (including rLM-linked hypopnea/apnea) were defined as respiratory events associated with rLM.

**Figure 1.**
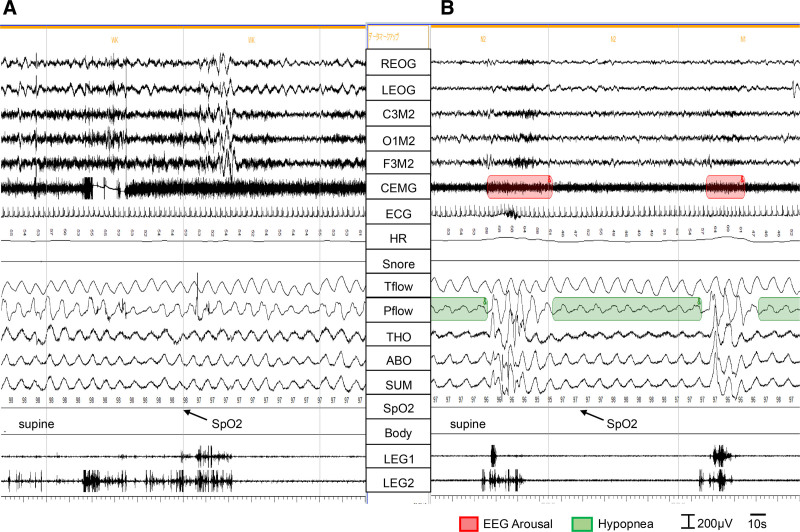
Respiratory flow signal during wakefulness (A: baseline) and during a hypopnea event under sleep conditions (B). (B) A hypopnea event lasting more than 10 seconds is observed, surrounded by arousal events accompanied by leg movements. Given the absence of a noticeable difference in P-flow (pressure at nasal cannula) between the baseline and the hypopnea event, it can be concluded that the hypopnea is not associated with increased inspiratory flattening. Furthermore, during the hypopnea event, neither snoring nor thoracoabdominal paradox is observed, indicating that the hypopnea event is central in nature. (B) The subject case is Case 2. Sleep stage is N1 & N2. Sampling rates of 100 Hz for TFlow, CanFlow, THO RIP, and ABD RIP. ABO = abdominal RIP, Body = body position, CEMG = chin electromyogram, ECG = electrocardiogram, HR = heart rate, LEG1 and LEG2 = electromyogram of right leg and left leg, P-flow = pressure at nasal cannula, REOG and LEOG = right and left electrooculogram, RIP = respiratory inductance plethysmography, SpO_2_ = oxygen saturation by pulse oximetry, SUM = RIP sum, TFlow = thermal flow, THO = thoracic respiratory inductance plethysmography.

Central and obstructive hypopneas (CH and OH, respectively) were classified as follows: Hypopneas were scored as obstructive if any of the following criteria were met: (a) snoring during the event, (b) increased inspiratory flattening of nasal pressure compared to baseline breathing, or (c) an associated thoracoabdominal paradox during the event but not during pre-event breathing. Hypopneas were scored as central if none of these criteria were met (Fig. 1 ).

The period from the start of PSG with lights off to the interruption for acuinjection with lights on was defined as the pre-acuinjection period (Pre-a). The post-acuinjection period (Post-a) was from lights off at PSG recommencement to the reoccurrence of LMs. LMs were considered to have reoccurred if they appeared multiple times within 5 minutes.

The period from the resumption of PSG after the acuinjection to the end of it, distinct from Post-a, was defined as restarted PSG. In cases where PAP-titration PSG was performed during the latter part of the overnight PSG, the restarted PSG period excluded PAP-titration PSG.

At our clinic, written consent for acuinjection is obtained before PSG from all patients with suspected OSA in preparation for cases where acuinjection may become indicated. This study retrospectively analyzed PSG parameters from PSG records and included patients from August 1, 2019, to December 31, 2023.

Written informed consent for academic use of clinical data was obtained from all 3 patients. The study was approved by the Ethics Committee of Fukuoka Sleep Clinic (approval number: 6) and conducted in accordance with the tenets of the Declaration of Helsinki.

## 3. Results

We examined 3 male patients with varying degrees of sleep disturbances and suspected sleep apnea.

### 3.1. Case 1

A 54-year-old male experienced frequent nighttime awakenings. Home sleep apnea testing indicated an oxygen desaturation index (number of desaturation events) ≥ 3%/h (ODI3%) of 9.3 events/h, and his Japanese version of the Epworth Sleepiness Scale (JESS) score was 6. The patient was advised by an occupational physician to undergo further examination for sleep apnea.

After the start of PSG on March 3, 2023, he fell asleep quickly but had frequent LMs (in Pre-a: LMI = 152.7 events/h), mainly in his right leg, associated with arousal.

At 65.9 minutes after the start of PSG, acuinjection was performed on ST36. During the acuinjection, the patient reported a warm and heavy sensation spreading downward from the injection site in both lower limbs.

**Figure 2.**
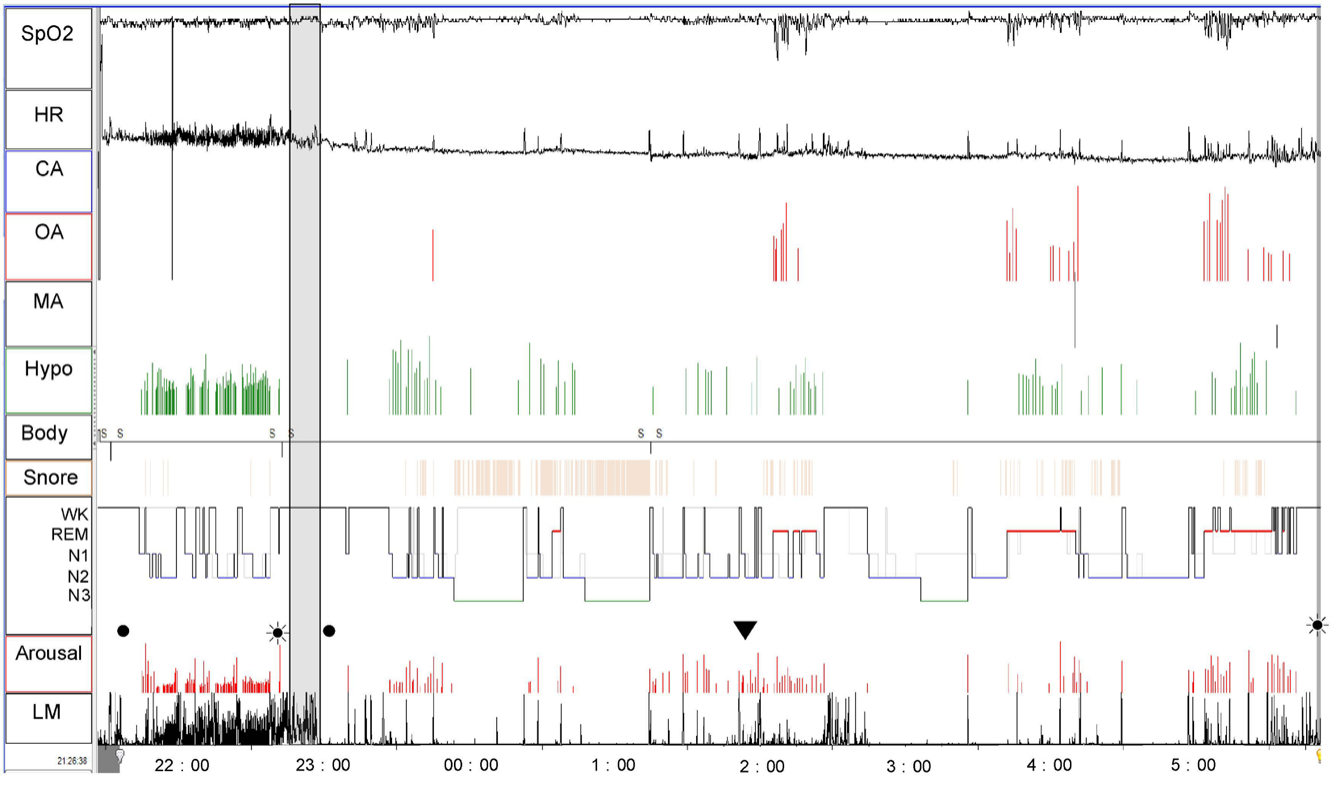
Polysomnography trend graph of Case 1. A 54-year-old male experienced frequent nighttime awakenings. Although the patient fell asleep after the start of polysomnography (PSG), he exhibited frequent LM (LMI = 152.7/h) and repeated EEG arousals (ArI = 133.6/h). Consequently, 65.9 minutes after the start of PSG, the lights were turned on, and after a brief reconfirmation of the explanation and consent, an acuinjection was performed at ST36. Following the acuinjection, LM was almost completely resolved; however, mild recurrence of LM – defined as multiple LM events within 5 minutes – was observed 176.9 minutes after the acuinjection. Even so, throughout the entire restarted PSG session (414.4 minutes), the frequency of LM was minimal, with an LMI of 14.0/h. Before the acuinjection, frequent central hypopnea associated with LM was observed (CHI = 57.3 events/h). However, during the entire restarted PSG session after the acuinjection, central hypopnea was almost completely resolved. Instead, the patient exhibited obstructive sleep apnea of moderate grading (AHI = 20.8, OAHI = 20.2) that worsened during REM sleep. Treatment involved an oral appliance (OA), with LM under follow-up observation. During the acuinjection, the patient reported experiencing deqi (a warm and heavy sensation spreading downward from the injection site in both lower limbs). (filled circle) light off; (filled circle surrounded by dots) light on; (down triangle) reoccurrence of LM after it was suppressed by acuinjection. The reoccurrence of LMs was defined as multiple occurrences of LMs within 5 minutes. AHI = apnea–hypopnea index, arousal = arousal as observed on electroencephalography (EEG, arousal/h), Body = body position, CA = central apnea(/h), CHI = central hypopnea index, HR = heart rate (bpm), Hypo = hypopnea (/h), LM = leg movement, LMI = leg movement index, MA = mixed apnea (/h), N1 = N1 stage of sleep, N2 = N2 stage of sleep, N3 = N3 stage of sleep, OA = obstructive apnea (/h), OAHI = obstructive apnea–hypopnea index, REM = R stage of sleep, SpO_2_ = oxygen saturation measured by pulse oximetry(%), WK = wake stage of sleep.

In the restarted PSG, the patient showed an AHI of 20.8 (obstructive AHI [OAHI] 20.2, central AHI [CAHI] 0.2, mixed apnea index 0.4) and an LMI of 14.0, indicating mild OSA with reduced LMs (Fig. 2).

The patient was recommended to use an oral appliance for treatment. However, according to the occupational physician’s report after the test, the patient experienced symptom improvement and did not proceed with treatment.

### 3.2. Case 2

A 39-year-old male had daytime sleepiness, nocturnal awakenings, and nocturnal urination. He had a respiratory disturbance index (RDI) of 19.5 events/h, an ODI3% of 18.7 events/h, and a JESS score of 16.

During PSG on June 29, 2021, he fell asleep quickly after the start of the PSG and entered Stage N3 sleep, during which frequent LMs occurred in both legs, but there was little wakefulness (Stage W).

**Figure 3.**
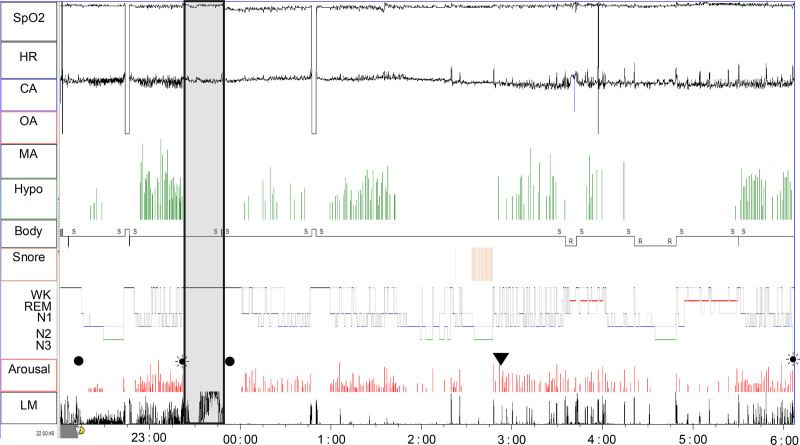
Polysomnography trend graph of Case 2. A 39-year-old male presented with daytime sleepiness, nocturnal awakenings, and nocturnal urination. During the period from the start of polysomnography (PSG) to the acuinjection (pre-a: 68.8 minutes), frequent LM occurred, including in the first half, during which no sleep fragmentation was observed, allowing stage N3 sleep to be achieved. However, after urinating 31 min after the start of PSG, frequent sleep fragmentation associated with LM was observed. In the latter half of the pre-a period, LM was accompanied by central hypopnea, with the overall LMI for the pre-a period being 142.4 events/h and the CHI being 33.6 events/h. Immediately after the acuinjection, LM was almost completely suppressed; however, it recurred 184 minutes later (recurrence defined as multiple LM events within 5 min). Even so, throughout the entire restarted PSG session after the acuinjection, the frequency of LM remained low (LMI = 14.2), central hypopnea was not observed, and the patient exhibited OSA, characterized mainly by obstructive hypopnea, with an AHI of 20.0. CPAP therapy was prescribed, which improved his symptoms. During the acuinjection, the patient reported a heavy sensation spreading downward from the injection site in both legs. (filled circle) light off; (filled circle surrounded by dots) light on; (down triangle) reoccurrence of LM after it was suppressed by acuinjection. The reoccurrence of LMs was defined as multiple occurrences of LMs within 5 min. AHI = apnea–hypopnea index, Arousal = arousal as observed on electroencephalography (EEG, arousal/h), Body = body position, CA = central apnea (/h), CHI = central hypopnea index, CPAP = continuous positive airway pressure, HR = heart rate (bpm), Hypo = hypopnea (/h), LM = leg movement, LMI = leg movement index, MA = mixed apnea (/h), N1 = N1 stage of sleep, N2 = N2 stage of sleep, N3 = N3 stage of sleep, OA = obstructive apnea (/h), REM = R stage of sleep, SpO_2_ = oxygen saturation measured by pulse oximetry (%), WK = wake stage of sleep.

However, after urinating 31 minutes after the start of PSG, the subsequent PSG showed frequent LMs (in Pre-a: 142.4 events/h) and repeated awakenings. Thus, 68.8 minutes after the start of PSG, acuinjection was performed on ST36, leading to the prompt suppression of frequent LMs and arousals. During acuinjection, the patient reported a heavy sensation spreading downward from the injection site in both legs. In the restarted PSG, the patient had an AHI of 20.0 (OAHI = 16.4, CAHI = 3.6) and an LMI of 14.2 (Fig. 3).

Continuous PAP (CPAP) therapy was prescribed, which improved his symptoms.

### 3.3. Case 3

A 42-year-old male presented with complaints of apnea episodes and poor recovery from fatigue. His RDI was 33.9 events/h, his ODI3% was 30.6/h, and his JESS score was 19. During PSG conducted on January 28, 2020, frequent LMs (LMI = 129.6), including PLMS (PLMI = 67.0) and rLM (rLMI = 37.4), were observed during the Pre-a period. Furthermore, frequent EEG arousals caused by respiratory events and LMs were noted, with a respiratory arousal index (Resp-ArI) of 43.5 and an LM arousal index (LM-ArI) of 41.7.

Acuinjection therapy was performed at ST36, 95.5 minutes after the start of the PSG. As a result, in the Post-a period, the LMI was remarkably suppressed to 6.6 (PLMI = 0, rLMI = 0.7).

The restarted PSG ended at 4:30 am, and PAP-titration PSG started at 4:43 am, confirming the effectiveness of CPAP with an AHI of 0 events/h.

**Figure 4.**
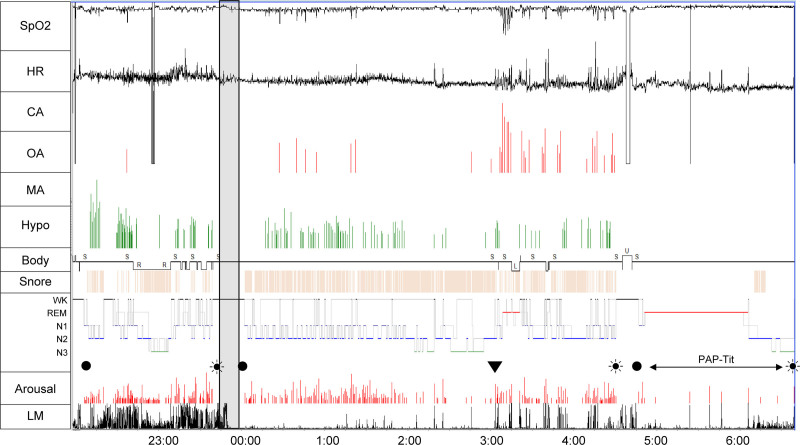
Polysomnography trend graph of Case 3. A 42-year-old male reported apnea episodes and poor recovery from fatigue. During the period from the start of polysomnography (PSG) to the acuinjection (Pre-a: 95.5 min), the LMI was 129.6 events/h, respiratory-related LM was 37.4, and the periodic leg movement in sleep (PLMS) index was 67.0, indicating a high proportion of PLMS among LM events. Additionally, the respiratory EEG-arousal (RespAr) index and LM-related EEG-arousal (LMAr) index were 43.5/41.7, showing that both snoring-associated respiratory events and LM frequently caused EEG arousals. Midway through the Pre-a period, stage N3 sleep was achieved in the lateral position; however, once in the supine position, frequent sleep fragmentation occurred. After restarting PSG following the acuinjection, LM, including PLMS, was almost completely resolved. However, LM mildly recurred 193.6 min after the restarted PSG (recurrence defined as multiple LM events within 5 min). The restarted PSG ended at 4:30 am, and PAP-titration PSG started at 4:43 am Observations throughout the restarted PSG session showed that LM was largely suppressed (LMI = 11.9), and OSA with snoring was demonstrated (AHI = 29.3). Effective CPAP therapy (AHI = 0 events/h) was confirmed during PAP-titration PSG, indicating its use. (filled circle) light off; (filled circle surrounded by dots) light on; (down triangle) reoccurrence of LM after it was suppressed by acuinjection. The reoccurrence of LMs was defined as multiple occurrences of LMs within 5 min. AHI = apnea–hypopea index, Arousal = arousal as observed on electroencephalography (EEG, arousal/h), Body = body position, CA = central apnea (/h), CPAP = continuous positive airway pressure, HR = heart rate (bpm), Hypo = hypopnea (/h), LM = leg movement, LMI = leg movement index, MA = mixed apnea (/h), N1 = N1 stage of sleep, N2 = N2 stage of sleep, N3 = N3 stage of sleep, OA = obstructive apnea (/h), PAP-Tit = positive airway pressure-titration, REM = R stage of sleep, SpO_2_ = oxygen saturation measured by pulse oximetry (%), WK = wake stage of sleep.

In the restarted PSG, the AHI was 29.3 (OAHI = 29.3, CAHI = 0.0) and the LMI was 11.9 (Fig. 4). CPAP therapy was indicated, which subsequently improved his recovery from fatigue. An overview of the 3 patients and their responses to acuinjection is presented in Table 1, which summarizes their characteristics, chief complaints, and responses to treatment.

**Table 1:** Overview of patients and response to acuinjection.

Case		Case 1	Case 2	Case 3
Age (yr)/sex		54/male	39/male	42/male
HSAT before PSG, events/h		ODI3% = 9.3	RDI = 19.5, ODI3% = 18.7	RDI = 33.9, ODI3% = 30.6
Chief complaint		Frequent nighttime awakenings	EDS and nocturnal awakenings	Poor recovery from fatigue
JESS score		6	16	19
AHI/LMI (ArI), events/h	Before acuinjection	125.5/ 152.7 (133.6)	47.1/ 142.4 (62.8)	45.2/ 129.6 (103.5)
	After acuinjection	20.8/ 14.0 (20.7)	20.0/ 14.2 (40.8)	29.3/ 11.9 (49.1)
Treatment prescribed after PSG		OA	CPAP	CPAP
Treatment outcome after prescription therapy		Symptoms improved without the use of OA	EDS, nocturia and nocturnal awakenings improved	Fatigue recovery was achieved

The term “before acuinjection” refers to the period from the start of PSG to the time of acuinjection at ST36, corresponding to “pre-a” in the text, while “after acuinjection” denotes the PSG session that was restarted following acuinjection, excluding CPAP titration studies, and corresponds to “restarted PSG” in the text. Acuinjection was performed bilaterally at ST36, with pentazocine (3.75 mg in 0.5 mL NS or 7.5 mg in 1.0 mL NS) administered at each site.

AHI = apnea–hypopnea index, events per hour, ArI = electroencephalography arousal index, events per hour, CPAP = continuous positive airway pressure, EDS = excessive daytime sleepiness, HSAT = home sleep apnea testing, JESS = Japanese version of the Epworth Sleepiness Scale, LMI = leg movement index, events per hour, OA = oral appliance, ODI3% = oxygen desaturation index with ≥3% desaturation, events per hour, PSG = polysomnography, RDI = respiratory disturbance index, events per hour.

All 3 patients exhibited no abnormalities in the physical examination conducted by the physician and no findings in the lower extremities, chest radiographs, electrocardiograms, or blood biochemistry. Acuinjection therapy at the bilateral ST36 acupoint effectively and promptly resolved LMs, enabling uninterrupted sleep and facilitating subsequent studies. No notable acuinjection-related complications were observed.

### 3.4. Overview of PSG

The acuinjection interruption of PSG lasted an average of 18.0 minutes across the 3 cases. Post-a lasted 184.8 minutes. LMs were almost completely suppressed immediately after acuinjection. The restarted PSG lasted 357.6 minutes. The Pre-a lasted 76.7 minutes.

In the Pre-a, the overall AHI was 72.6/h, primarily composed of hypopneas (hypopnea index: 72.3/h), with minimal apneas (apnea index: 0.3/h). Notably, the mean OAHI was 39.4 events/h, and the CAHI was 33.2 events/h. These findings indicate severe OSA, with central events contributing nearly half of the total AHI.

Frequent rLMs were observed at a rate of 68.4 events/h, potentially contributing to sleep fragmentation.

In Post-a, the mean AHI decreased to 19.9/h and the hypopnea index to 18.9/h, with hypopneas remaining predominant. Despite this clear reduction in AHI, the minimum oxygen saturation decreased slightly, from 91.3% Pre-a to 88.3% Post-a (Table 2). This decline in oxygen saturation, despite the improvement in AHI, is likely attributable to changes in the pattern of respiratory events, specifically a reduction in rLM-related hypopneas, which are associated with less severe desaturation.

**Table 2:** PSG parameters of the 3 patients.

Items	Mean (n = 3)		
Age (yr)	45 (39–54)		
Sex	M: 3		
BMI (kg/m^2^)	24.8 (22.1–27.0)		

Observation phase	Pre-a	Post-a	Restarted PSG
TRT (min)	76.7 (65.9–95.5)	184.8 (176.9–193.6)	357.6 (280.3–414.4)
AHI (/h)	72.6 (45.2–125.5)	19.9 (15.1–24.8)	23.4 (20.0–29.3)
AI/ HI (/h)	0.3 (0.0–9.0)/ 72.3 (44.3–125.5)	1.1 (0.0–2.8)/ 18.9 (14.7–22.0)	4.8 (0.2–8.0)/ 18.6 (14.6–21.3)
OAHI (/h)	39.4 (13.5–68.2)	19.8 (14.6–24.8)	22.0 (16.4–29.3)
OAI/ OHI (/h)	0.3 (0.0–0.9)/ 39.1 (13.5–68.2)	1.1 (0.0–2.8)/ 18.7 (14.2–22.0)	4.6 (0.0–8.0)/ 17.4 (14.4–21.3)
CAHI/ mixed AI (/h)	33.2 (8.7–57.3)/ 0	0.1 (0.0–0.4)/ 0	1.3 (0.0–3.6)/ 0.1 (0–0.4)
CAI/ CHI (/h)	0/ 33.2 (8.7–57.3)	0/ 0.1 (0.0–0.4)	0.1 (0.0–0.2)/ 1.2 (0.0–3.4)
LMI (/h)	141.6 (129.6–152.7)	5.9 (2.8–8.2)	13.4 (11.9–14.2)
rLMI (/h)	68.4 (37.4–124.1)	0.7 (0.0–1.3)	5.3 (4.6–6.4)
NrLMI (/h)	73.2 (28.6–98.7)	5.2 (2.8–6.9)	8.1 (7.3–9.0)
PLMSI (/h)	49.2 (5.5–75.1)	0	2.1 (1.9–2.3)
ArI/ Spont-ArI (/h)	100.0 (62.8–133.6)/ 8.5 (2.7–18.3)	36.7 (20.6–45.9)/ 16.5 (7.3–22.8)	36.9 (20.7–49.1)/ 14.0 (5.8–18.9)
PLMS ArI (/h)	13.6 (1.4–30.4)	0	0.6 (0.0–1.3)
Resp-ArI/ LM-ArI (/h)	70.7 (43.5–122.7)/ 20.7 (8.2–41.7)	17.1 (9.9–22.4)/ 3.0 (1.6–4.1)	18.7 (10.3–26.1)/ 4.2 (3.8–4.7)
ODI3% (/h)/ Min-SpO_2_ (%)	13.7 (3.4–28.8)/ 91.3 (88.0–95.0)	10.0 (8.6–12.0)/ 88.3 (85.0–92.0)	12.5 (7.7–17.8)/ 83.3 (79.0–91.0)
SE (%)	72.3 (66.8–77.8)	83.0 (78.9–89.9)	84.3 (80.5–88.5)
REM sleep			2.3 (1.0–4.0)
Heart rate (average, bpm)	69.7 (55.5–82.0)	64.0 (57.6–75.7)	61.4 (54.8–74.4)

The data are presented as mean (min–max).

AHI = apnea–hypopnea index, AI = apnea index, ArI = EEG-arousal index, BMI = body mass index, CAHI = central apnea–hypopnea index, CAI = central apnea index, CHI = central hypopnea index, h = hour, HI = hypopnea index, index = number of events/h, LM-ArI = leg movement-related EEG-arousal index, LMI = leg movement index, Min-SpO_2_ = minimum SpO2 (saturation of peripheral oxygen) during sleep, NrLMI = the index of LMs that are not respiratory-related leg movements, OAHI = obstructive apnea–hypopnea index, OAI = obstructive apnea index, ODI3% = the oxygen desaturation index ≥3% (number of desaturation events ≥3% per hour), OHI = obstructive hypopnea index, PLMS = periodic leg movement in sleep, PLMS ArI = PLMS-related EEG-arousal index, PLMSI = PLMS index, Post-a = The period from light-off after acuinjection to the appearance of multiple LMs per 5 minutes, Pre-a = the period from lights off to lights on prior to acuinjection, Resp-ArI = respiratory event-related EEG-arousal index, Restarted PSG = polysomnography restarted after acuinjection, rLMI = respiratory-related LM index, SE = percentage sleep efficiency (sleep time as percentage of recording time, [total sleep time/time in bed] ×100), Spont-ArI = ArI excluding Resp-ArI and LM-ArI, TRT = total recording time in min.

### 3.5. Changes in PSG parameters

Acuinjection reduced the LMI by 95.8% (141.6 to 5.9/h) and rLM by 99.0% (68.4 to 0.7/h). PLMS were eliminated in Post-a. LM expression was absent immediately after acuinjection but reoccurred at the end of Post-a.

Most respiratory events in the Pre-a (Pre-a AHI of 72.6/h) were rLM-linked apnea–hypopnea, with 68.4/h being rLM-linked. Of these, apnea was 0.3/h, and hypopnea was 68.1/h. CH accounted for 48.3% (32.9/h) and OH for 51.7% (35.2/h).

**Figure 5.**
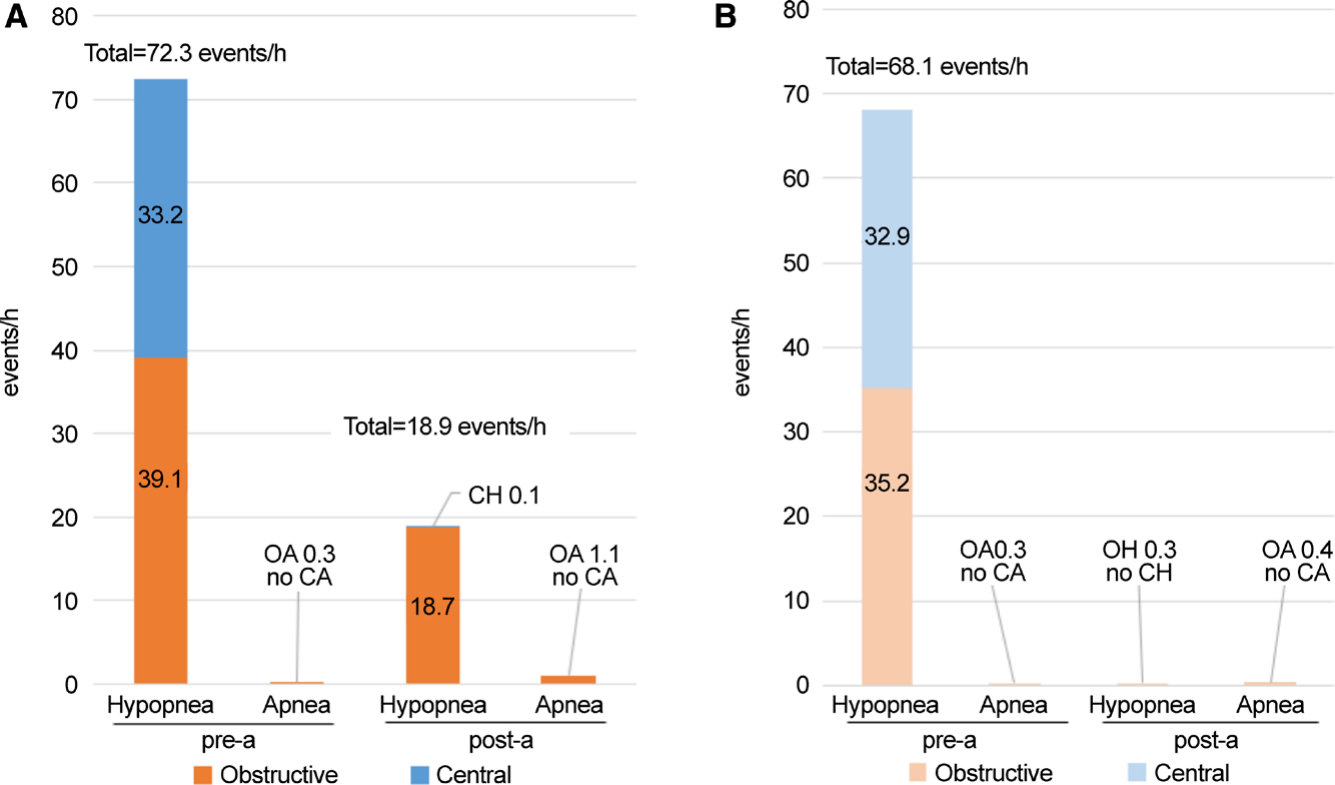
The impact of acuinjection on respiratory event frequency and type classification. The panels represent the average values across all 3 patients, with panel (A) showing the total respiratory events and panel (B) illustrating the rLM-linked respiratory events (respiratory events associated with respiratory-related leg movements). Among the total respiratory events shown in panel (A), 94.2% (68.4/72.6 events/h) are composed of rLM-linked respiratory events. When focusing on rLM-linked respiratory events (panel B), during the Pre-a period, apnea events are rare (0.3 events/h) and are predominantly composed of hypopneas. Of these hypopneas, 48.3% (32.9/68.1) are central hypopneas, while obstructive hypopneas account for 51.7% (35.2/68.1). In the Post-a period following the acuinjection, central apnea/hypopneas disappear completely; however, there remains a trace presence of obstructive hypopnea (0.3 events/h) and obstructive apnea (0.4 events/h). In contrast, when looking at the total respiratory events (panel A), although central apnea/hypopneas disappear in the Post-a period, obstructive hypopneas persist at 18.7 events/h and do not disappear. It is possible that the obstructive hypopnea events persisting in the Post-a period correspond to obstructive hypopnea events that existed as rLM-linked respiratory events during the Pre-a period. Pre-a: pre-acupuncture injection period, Post-a: post-acuinjection period.

From Pre-a to Post-a, the CHI dropped from 33.2 to 0.1 events/h, primarily driven by a reduction in LM-related central hypopneas, which decreased from 32.9 to 0 events/h. This demonstrates the remarkable efficacy of acuinjection in suppressing CH (Fig. 5). However, the OHI decreased from 39.1 to 18.7, with a residual obstructive apnea index of 1.1. Despite the disappearance in CHI, OH persisted.

The mean AHI decreased from 72.6/h to 19.9/h, and the mean EEG-arousal index (ArI) decreased from 100.0/h to 36.7/h. When Spont-ArI is defined as ArI excluding Resp-ArI and LM-ArI, it increased from 8.5 in Pre-a to 16.5 in Post-a.

In the restarted PSG, LMI showed a slight increase from Post-a (5.9–13.4), while ArI and CHI remained low with minimal increases (ArI: 36.7 to 36.9, CHI: 0.1 to 1.2).

## 4. Discussion

The 3 cases presented in this study exhibited excessive daytime sleepiness and nocturnal awakenings, requiring precise diagnosis of sleep apnea based on home sleep apnea testing results. During the Pre-a period (mean 76.7 minutes), all 3 cases showed frequent LMs (LMI = 141.6 events/h) and OSA with frequent central hypopneas (AHI = 72.6, OAHI = 39.4, CHI = 33.2 events/h), and sleep was fragmented.

Acuinjection was performed bilaterally at ST36. As a result, during the Post-a period (184.8 minutes), LMs were remarkably suppressed (LMI = 5.9 events/h). Furthermore, compared to the frequency of CH and OH during the Pre-a period (CHI: 33.2, OHI = 39.1), the frequency in the Post-a period was clearly reduced (CHI: 0.1, OHI: 18.7), suggesting that LMs may promote the occurrence of hypopnea, particularly central hypopnea. This demonstrates that acuinjection at ST36 is effective in diagnosing OSA, free from the influence of LMs.

### 4.1. Effects of acuinjection on LM

Following acuinjection at ST36, LMs were effectively suppressed (LMI: 141.6 in Pre-a vs 5.9 in Post-a), allowing for continuous PSG without frequent sleep fragmentation caused by LMs.

Without acuinjection, 2 patterns of nighttime PLMS distribution have been reported^[12]^: 1 with high activity upon falling asleep that decreases throughout the night, and another with relatively consistent activity across the night. However, in these 3 cases, LMs, including PLMS, were suppressed immediately after acuinjection for more than 184.8 minutes, showing a different pattern from the usual nighttime PLMS distribution. This suggests that the decrease in Post-a LMs is attributable to acuinjection rather than nighttime patterns or other sleep conditions, including sedated sleep.

The effect of acuinjection is rapid, and as seen in Case 3, LMs were resolved during sleep immediately after the injection. Therefore, it can be concluded that the effect of acuinjection on LM was not due to the pharmacological action of pentazocine included in the solution.

### 4.2. Effects of acuinjection on rLM-linked hypopnea

In all 3 cases, hypopneas accounted for 99.6% of respiratory events in Pre-a (HI/AHI = 72.3/72.6) and 95.0% in Post-a (18.9/19.9). This confirms that the observed OSA cases were primarily hypopnea-driven (Table 2, Fig. 5). In Pre-a, 94.2% of hypopneas were rLM-linked (total hypopnea vs rLM-linked hypopnea: 72.3 vs 68.1 events/h), with 32.9 events/h being CH. All CH events were effectively suppressed by acuinjection, indicating that CH is highly responsive to acuinjection. Similarly, in Pre-a, most OH was rLM-linked (total vs rLM: 39.1 vs 35.2). However, in Post-a, although rLM-linked OH resolved, non-rLM-linked OH (OH other than rLM-linked OH) emerged at 18.7 events/h. In Pre-a, LM may have facilitated the formation of rLM-linked OH (Fig. 5).

### 4.3. Effects of acuinjection on EEG arousals

The ArI decreased markedly with acuinjection (Pre-a: 100.0/h vs Post-a: 36.7/h), likely due to reduced LMs. PLMS with arousals are associated with a higher risk of non-sustained ventricular tachycardia,^[13]^ highlighting the potential clinical relevance of targeting such arousals.

Pramipexole suppresses PLMS but does not reduce EEG arousals in patients with RLS,^[10]^ indicating its potential limitation. A cited study evaluating the effects of 0.25 mg oral pramipexole on PLMS index (PLMSI) and ArI in patients with RLS (n = 17) using overnight PSG reported the following results: PLMSI decreased from 42.7 ± 32.2 to 6.0 ± 8.3 (*P* < .003), while ArI showed no significant change (51.8 ± 34.3 vs 45.1 ± 30.0, *P* > .05).

In contrast, our study demonstrated that acuinjection completely eliminated PLMSI (49.2 Pre-a to 0 Post-a). Spont-ArI, including dissociated EEG arousals when present, showed a slight increase (8.5 Pre-a to 16.5 Post-a). These results suggest that acuinjection does not induce dissociated EEG arousals from PLM or, if it does, their frequency is minimal. However, sleep quality after overnight PSG was not assessed, and thus, the clinical impact of reducing PLMS-related arousals remains undetermined.

The reduction in rLM (rLMI: 68.4 Pre-a to 0.7 Post-a) was accompanied by decreased EEG arousals associated with rLM-linked respiratory events. As with PLMS-related arousals, the clinical significance of this reduction warrants further investigation.

### 4.4. Proposed mechanism of acuinjection in suppressing LMs

Although the mechanism of rLM is not well understood, it shares similarities with PLMS.^[14]^ Key factors in PLMS include spinal cord and sympathetic nerve overactivation. These key factors are discussed briefly below.

#### 4.4.1. Spinal cord overactivation

Increased spinal cord excitability in PLMS has been suggested by observations of thoracic spinal cord lesions^[15]^ and electrically induced spinal flexor reflexes.^[16]^

Acupuncture suppresses spinal cord hyperexcitability in conditions like post-traumatic brain injury,^[17]^ likely through pathways involving tumor necrosis factor-α and interleukin-1β inhibition.^[18]^ This suggests that acupuncture reduces LMs by mitigating spinal cord overactivation.

#### 4.4.2. Sympathetic overactivation

Sympathetic overactivation is considered a key factor in PLMS, as evidenced by findings in heart rate variability and EEG spectral changes^[19]^ and increased muscle sympathetic nerve activity during PLMS.^[20]^ Similar overactivation is also observed in rLM.^[19]^

Acupuncture at ST36 suppresses sympathetic pathways activated by chronic stress^[21]^ and inhibits sympathetic-sensory coupling in the dorsal root ganglion,^[22]^ which amplifies sensory input and inflammation.^[23]^ By suppressing sympathetic activation, acupuncture may inhibit LM occurrence.

### 4.5. Proposed mechanism of acuinjection in suppressing EEG-arousal

Arousal operates within a hierarchical structure.^[24]^ Thus, the influence of acupuncture on arousal may not follow a single pathway. LMs are not primary but rather a phenomenon associated with an underlying arousal disorder.^[25]^ Therefore, understanding arousals related to LMs is crucial for comprehending LM pathology.

Cortico-thalamo-cortical interaction modulates EEG-arousal.^[26]^ Interestingly, in monkeys, electroacupuncture suppresses pain-induced activation in brain areas, including the thalamus, achieving analgesia.^[27]^ Similarly, arousals associated with LM may be modulated via thalamocortical circuits and suppressed by acuinjection, akin to the modulation of pain.

### 4.6. Proposed mechanism of acuinjection in suppressing hypopnea

The transition between wake and sleep can induce state instability, causing central apneas.^[28]^ Furthermore, arousals exacerbate upper airway collapse by increasing post-apneic hyperventilation.^[29]^

Since acuinjection suppresses EEG arousals, it is hypothesized that LM-associated hypopneas, including CH and OH, are alleviated or resolved through acuinjection.

### 4.7. Clinical significance of acuinjection at ST36

In cases of PLMS, the impact of non-ventilatory variables on sleep apnea has been noted.^[6]^ Our cases demonstrated that suppressing LM with acuinjection resolves LM-linked hypopnea, supporting previous observations.^[6]^

A poor relationship exists between daytime symptoms such as sleepiness and the severity measured by the AHI.^[29]^ The intervention on LM and its impact on AHI in our cases may partly explain this poor relationship.

Acuinjection at ST36 suppresses sleep fragmentation and EEG-arousal associated with LMs, enabling PSG studies to be less influenced by LMs. Acuinjection administered at anatomically safe sites – such as ST36, which is located away from major organs or neurovascular structures – has not been associated with clinically significant complications, apart from the general risk of infection inherent to any skin puncture.^[30]^ This site is considered suitable for acuinjection from both a therapeutic and safety standpoint. It is a simple method that yields rapid effects on LM. Thus, it may be useful in the treatment and research of LMs.

However, it has the following drawbacks:

- LM data after the acuinjection are lost.
- If acuinjection is performed during a PSG study, mild pain must be inflicted at night for the acuinjection.
- Performing acuinjection imposes a burden on the attending physician (in Japan, acuinjection is legally restricted to medical doctors).

Furthermore, the issue of whether PSG results under acuinjection are suitable for evaluating the sleep and breathing of OSA remains unverified and requires further investigation.

### 4.8. About acuinjection

Acuinjection is an acupoint-stimulating technique that involves injecting liquid agents instead of using needle-based acupoint manipulation. Needle acupuncture in traditional Chinese medicine requires needle manipulations such as insertion, twisting, and withdrawal. However, acuinjection is a simple injection technique, making it easier to perform for Western physicians.

### 4.9. Three-dimensional identification of the ST36 acupoint and its clinical implications

With the knee flexed at 90°, ST36 is located at the center of the depression lateral to the tibial tuberosity, approximately 1 finger-breadth (middle finger) below the upper edge of the depression, as described in the *Acupuncture Text* jointly edited by Japan and China.^[31]^

According to the same text,^[31]^ a straight needle is typically inserted 0.5 to 1 F-cun (thumb-width) into ST36. In Japanese adults, 1 F-cun corresponds to approximately 2.0 − 2.2 cm.^[32]^ Thus, the estimated needling depth was 1.0 − 2.2 cm, and in this study, it was set at approximately 1.5 cm.

Although traditional methods offer only an approximate location, *deqi* – a characteristic sensation such as heaviness or numbness – suggests accurate needle placement.

Notably, even when the injection site deviates slightly from the surface location or remains subcutaneous, therapeutic effects are still observed. Therefore, pinpoint accuracy is not essential.^[11]^

### 4.10. Volume of acuinjection solution

To reduce injection-related pain, Watanabe recommends administering a small volume of 0.01 to 0.25 mL.^[11]^ However, due to anatomical variations, a volume exceeding 0.25 mL may enable broader diffusion and enhance stimulation.

In 2 cases in our study, *deqi* occurred after more 0.25 mL of a 0.5 mL or 1.0 mL injection was administered, indicating that a larger volume may be effective; however, the role of infiltration time cannot be ruled out.

In our study, no pain was reported aside from needle insertion. The use of 1.0 mL of NS for ST36 acuinjection has also been previously reported,^[33]^ supporting its appropriateness. The 0.5 mL used in Case 1 may represent an intermediate step toward the 1.0 mL volume employed here. Further investigation is warranted to determine the optimal dose.

### 4.11. Complications of acuinjection at ST36

Although the overall clinical risk of acuinjection at ST36 is considered low, evidence specific to its safety remains limited. A review of 4 randomized controlled trials (267 patients) evaluating ST36 injection for diabetic peripheral neuropathy using mecobalamin, vitamin B1, or B12 reported no serious adverse events. The incidence of adverse events was similar to that in control groups receiving the same medications via intramuscular or intravenous routes (relative risk 1.86).^[34]^ However, the small sample sizes and low methodological quality of these studies limit the strength of the evidence.

The contraindications for acupuncture defined by the World Health Organization^[35]^ may be applied to acuinjection at ST36. These include:

1. Pregnancy: Acupuncture is typically avoided during the first trimester of pregnancy.^[36]^
2. Medical emergencies and surgical conditions
3. Malignant tumors: Acupuncture should not be used for the treatment of malignant tumors.
4. Bleeding disorders
5. Infected skin regions

Acupuncture and acuinjection have increasingly been studied beyond traditional Chinese medicine. ST36 acuinjection may hold therapeutic potential for LMs, LM-related respiratory events, and EEG arousals and may also contribute to understanding their clinical relevance.

### 4.12. Limitations

This study had several limitations. While the primary focus was on OSA cases with hypopnea events, it included a limited number of rLM-linked apnea events.

The LM suppression effect was observed over a single night without evaluating long-term effects. Additionally, sleep quality and subjective satisfaction were not assessed. Furthermore, the clinical significance of PSG findings during LM-suppressed sleep induced by acuinjection at ST36 was not examined.

Finally, the small sample size of 3 cases limits the generalizability of the findings.

## 5. Conclusions

Our study found that acuinjection at ST36 in patients with OSA and LMs suppressed LMs (including PLMS and rLM). This suppression was associated with the elimination of central apneas or the reduction of obstructive hypopneas linked to rLM, as well as the suppression of EEG arousals. Acuinjection at ST36 appears to be a promising approach for treating or researching LM.

## Acknowledgments

We thank Editage (https://www.editage.com/) for critically reviewing this manuscript.

## Author contributions

**Study conception and design:** Anjyu Minami, Takero Fukutome.

**Data collection:** Anjyu Minami, Haruna Minami, Harumi Matsuoka.

**Analysis and interpretation of results:** Anjyu Minami, Haruna Minami, Harumi Matsuoka, Takero Fukutome.

**Draft manuscript preparation:** Anjyu Minami, Takero Fukutome.

**Writing – review & editing:** Anjyu Minami, Haruna Minami, Harumi Matsuoka, Takero Fukutome.
